# Factors associated with the response to atezolizumab/bevacizumab combination therapy for hepatocellular carcinoma

**DOI:** 10.1002/jgh3.12932

**Published:** 2023-06-15

**Authors:** Yoshihiko Yano, Atsushi Yamamoto, Takuya Mimura, Saeko Kushida, Seiya Hirohata, Seitetsu Yoon, Hirotaka Hirano, Soo Ki Kim, Yuri Hatazawa, Kenji Momose, Hiroki Hayashi, Takuo Kado, Katsuhisa Nishi, Hidenori Tanaka, Takanori Matsuura, Ryutaro Yoshida, Naoki Asaji, Eiichiro Yasutomi, Yuuki Shiomi, Akihiro Minami, Shohei Komatsu, Takumi Fukumoto, Yoshihide Ueda, Yuzo Kodama

**Affiliations:** ^1^ Division of Gastroenterology Kobe University Graduate School of Medicine Kobe Japan; ^2^ Hyogo Prefectural Hyogo Cancer Center Akashi Japan; ^3^ Hyogo Prefectural Kakogawa Medical Center Kakogawa Japan; ^4^ Yodogawa Christian Hospital Osaka Japan; ^5^ Kobe Asahi Hospital Kobe Japan; ^6^ Konan Medical Center Kobe Japan; ^7^ Osaka Saiseikai Nakatsu Hospital Osaka Japan; ^8^ Kitaharima Medical Center Ono Japan; ^9^ Akashi Medical Center Akashi Japan; ^10^ Hyogo Prefectural Awaji Medical Center Sumoto Japan; ^11^ Sanda City Hospital Sanda Japan; ^12^ Division of Hepato‐Biliary‐Pancreatic Surgery Kobe University Graduate School of Medicine Kobe Japan

**Keywords:** adverse event, hepatocellular carcinoma, immune checkpoint inhibitor

## Abstract

**Background and Aim:**

The purpose of this study was to analyze factors associated with the overall survival (OS) of atezolizumab/bevacizumab combination therapy for advanced hepatocellular carcinoma (aHCC). We also assessed the OS of patients with ineffective therapy and those who discontinued treatment owing to adverse events (AEs).

**Methods:**

This retrospective multicenter study involved 139 patients with aHCC who received atezolizumab/bevacizumab combination therapy between November 2020 and September 2022.

**Results:**

The median duration of treatment was 136.5 days, and the median observation period was 316 days. The overall response rate was 40%, and the disease control rate was 78% according to mRECIST criteria. Grade ≥2 AEs occurred in 63 patients (43%) and led to treatment discontinuation in 16 patients. Multivariate analysis revealed that treatment response and occurrence of grade ≥2 AEs after therapy, as well as low level of albumin‐bilirubin (ALBI) grade and low level of des‐gamma carboxy prothrombin (DCP) before therapy, were extracted as factors that contributed to OS. Log‐rank tests with the Kaplan–Meier method showed significant differences in OS among these factors. The OS of patients who discontinued owing to AEs was significantly shorter than that of other patients.

**Conclusion:**

Not only factors before therapy but also treatment response and the appearance of AEs are involved in OS for atezolizumab/bevacizumab combination therapy. Although the development of AEs also contributed to OS, appropriate management of AEs is important to avoid discontinuing treatment with this combination.

## Graphical abstract

Factors associated with OS of atezolizumab/bevacizumab combination therapy for HCC were analyzed. Treatment response and appearance of adverse events (AEs) as well as low DCP and ALBI grade 1/2a before therapy were involved in OS. Among patients with AEs, those who continued treatment were able to obtain OS prolongation.

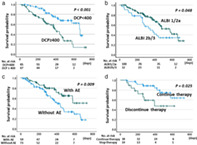

## Introduction

Atezolizumab/bevacizumab combination therapy is becoming established as a first‐line treatment for hepatocellular carcinoma (HCC).^1^, ^2^ Evidence suggests that the combination is safe to use and effective, even in elderly patients, because it has less severe side effects than conventional cytotoxic anticancer agents.^3^, ^4^ Recent real‐world studies have also reported its efficacy in patients with Child–Pugh B liver disease.^5^ In this multicenter study, the efficacy of atezolizumab/bevacizumab combination therapy was examined, and factors contributing to overall survival (OS) were analyzed.

On the other hand, immune checkpoint inhibitors (ICI) are associated with characteristic adverse events (AEs), which are reported to occur in approximately 20% of patients,^6^ and some patients do not achieve a therapeutic response because they stop treatment owing to AEs. However, some studies have shown that the appearance of AEs during treatment with an ICI is associated with their efficacy,^7^ and the implications of AE for treatment efficacy are not yet fully understood. In this study, the safety of atezolizumab/bevacizumab combination therapy and the impact of AE on treatment efficacy were also examined.

## Patients and methods

### Patients

The study included 139 patients (107 men, median age 74 years) who underwent atezolizumab/bevacizumab combination therapy for HCC at various institutions from November 2020 to September 2022 (Kobe University Hospital, Hyogo Prefectural Hyogo Cancer Center, Hyogo Prefectural Kakogawa Medical Center, Yodogawa Christian Hospital, Kobe Asahi Hospital, Osaka Saiseikai Nakatsu Hospital, Kitaharima Medical Center, Akashi Medical Center, Hyogo Prefectural Awaji Medical Center, and Sanda City Hospital). HCC was diagnosed based on an increasing trend of serum level of α‐fetoprotein (AFP) and/or des‐gamma carboxy prothrombin (DCP), as well as typical imaging findings and/or pathological findings.

Patients positive for hepatitis B virus surface antigen (HBsAg) or anti‐hepatitis C virus (HCV) were etiologically included in HCC due to hepatitis viral infection. patients with a history of alcohol abuse of 60 g/day or more were included as alcoholics.^8^

The treatment schedule was followed as approved regimens of atezolizumab and bevacizumab for advanced HCC. Both drugs were given at 21‐day intervals, with their continuation at the physician's discretion depending on AEs and patient's performance status.

As a rule, imaging evaluations were performed with contrast‐enhanced CT or MRI scans every 6–8 weeks, but intervals were allowed to vary according to the patient's condition and the attending physician's judgment. The treatment efficacy was evaluated according to the modified Response Evaluation Criteria in Solid Tumors (mRECIST) based on contrast‐enhanced computed tomography or contrast‐enhanced magnetic resonance imaging.^9^ AEs were assessed according to the National Cancer Institute Common Terminology Criteria for Adverse Events, version 5.0 (CTCAE version 5.0). According to the instructions, when grade 3 or more severe AEs occurred, dose reduction or temporary interruption was provided until symptoms subsided to pharmaceutically manageable grades 1 or 2.

This was a retrospective analysis of records stored in a database, and official approval was received based on the Guidelines for Clinical Research issued by the Ministry of Health and Welfare of Japan. This study was approved by the Ethics Committee of Kobe University Graduate School of Medicine and each participating institution (B200286).

### Statistical analysis

Factors associated with the efficacy of atezolizumab/bevacizumab combination therapy were analyzed using *t* tests and the log‐rank test with the Kaplan–Meier method to evaluate OS. To identify which factors significantly contributed to OS, Cox proportional hazards model was used for univariate and multivariate survival analysis. Statistical analyses were performed using SPSS version 28.

## Results

### Patient responses and characteristics of non‐responders

Overall, 133 patients were classified as Child–Pugh A, 102 as ALBI grade 1 or 2a, and 56 were within the up‐to‐seven criteria (Table 1). Esophageal varices were found in 43 patients, including after prophylactic hemorrhage treatment. The etiology comprised hepatitis viral infection in 75 patients (HBV in 27 and HCV in 48 patients, respectively), alcoholic cirrhosis in 43 patients, and nonalcoholic steatohepatitis in 19 patients. Comorbidities included hypertension in 81 patients and diabetes in 35 patients. One hundred and six patients had received prior therapy before atezolizumab/bevacizumab combination therapy, including chemotherapy in 51 patients.

**Table 1:** Comparison of clinical factors and outcomes between responders and non‐responders

	Total	Responders	Non‐responders	*P*
*N*	139	56	83	
Age (years)	73.1 ± 8.5	73.1 ± 8.5	73.2 ± 8.7	0.95
Sex (male)	107 (77%)	47 (84%)	60 (72%)	0.10
Etiology, viral hepatitis	75 (54%)	34 (61%)	41 (49%)	0.19
Etiology, alcohol	44 (32%)	17 (30%)	27 (33%)	0.79
BMI (kg/m^2^)	23.8 ± 7.2	23.8 ± 3.3	23.8 ± 8.8	0.98
Complication, hypertension	81 (58%)	32 (57%)	49 (59%)	0.83
Complication, diabetes	35 (25%)	12 (21%)	23 (28%)	0.40
Prior therapy	106 (76%)	37 (66%)	69 (83%)	0.02*
Prior chemotherapy	51 (37%)	9 (16%)	42 (51%)	<0.001*
Blood tests				
PLT (×10^3^/μL)	151 ± 66	142 ± 61	157 ± 68	0.19
AST (IU/L)	51.1 ± 34.9	54.5 ± 35.7	48.9 ± 35.3	0.36
ALT (IU/L)	36.4 ± 28.8	41.3 ± 35.4	33.0 ± 22.8	0.12
Albumin (g/dL)	3.75 ± 0.45	3.81 ± 0.41	3.71 ± 0.47	0.18
Prothrombin time (%)	90.5 ± 16.4	87.8 ± 18.0	92.2 ± 15.1	0.14
Total bilirubin (mg/dL)	0.91 ± 0.45	0.92 ± 0.43	0.90 ± 0.47	0.81
AFP > 400 ng/mL	45 (32%)	15 (27%)	30 (36%)	0.24
DCP > 400 mAU/L	68 (49%)	25 (45%)	43 (52%)	0.41
FIB4 index	4.14	5.03	4.96	0.92
Child–Pugh A	133 (96%)	56 (100%)	77 (93%)	0.04
ALBI score	−2.46	−2.52	−2.42	0.20
ALBI 1/2a	102 (73%)	45 (80%)	57 (69%)	0.12
Within up‐to‐seven criteria	56 (40%)	30 (54%)	26 (31%)	0.008
OS (days) (median)	321	403	309	0.004*
PFS (days) (median)	119	158	72	0.005*
Median treatment days (IQR)	136.5 (63–227.5)	175 (106–263)	78 (54–146)	<0.001*
Median treatment sessions (IQR)	6.0 (3.0–10.0)	5.0 (8.0–12.0)	4.0 (3.0–6.0)	<0.001*
Grade ≥2 AEs	59 (42%)	27 (48%)	32 (39%)	0.27
Discontinued treatment due to AE	16 (12%)	9 (16%)	7 (8%)	0.19
Post‐treatment	55 (40%)	25 (45%)	30 (36%)	0.32

^*^ Statistically significant.

Values are *n*, *n* (%), or mean ± standard deviation, unless otherwise specified.

AE, adverse event; AFP, alpha‐fetoprotein; ALBI, albumin–bilirubin; ALT, alanine aminotransferase; AST, aspartate aminotransferase; BMI, body mass index; DCP, des‐gamma‐carboxy prothrombin; FIB4, fibrosis‐4; IQR, interquartile range; n.s., not significant; OS, overall survival; PLT, platelet.

**Figure 1:**
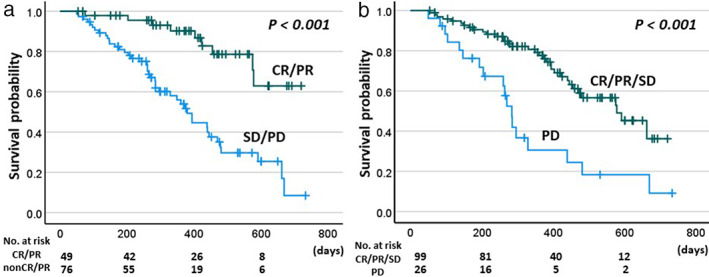
Kaplan–Meier plots of overall survival according to the treatment response. Overall survival was compared between patients with CR/PR and patients with SD/PD (non‐CR/PR) (a), and between patients with CR/PR/SD and patients with PD (b). CR, complete response; PD, progressive disease; PR, partial response; SD, stable disease.

The median duration of treatment was 136.5 days (IQR: 63–227.5 days, range: 20–601 days), median number of treatment sessions was 6.0 cycles (IQR: 3.0–10.0 cycles, range: 1–28), and the observation period ranged from 23 to 733 days (median 316 days). The best response, based on mRECIST, was complete response (CR) in 6 patients, partial response (PR) in 48 patients, stable disease (SD) in 55 patients, and progressive disease (PD) in 30 patients. Therefore, the overall response rate (ORR) was 40%, and the disease control rate (DCR) was 78%. The log–rank test with the Kaplan–Meier method revealed that patients with ORR and DCR experienced a survival benefit compared with the other patients (Fig. 1). The patients were therefore classified as either responders (patients who achieved CR or PR) or non‐responders (patients with SD or PD), and the characteristics of these two groups were compared, as summarized in Table 1. The responders included greater proportions of patients who had received prior therapy, including chemotherapy, and who met the up‐to‐seven criteria. The responders also received atezolizumab/bevacizumab combination therapy for longer and had a survival benefit compared with the non‐responders.

We also investigated possible factors associated with treatment response. Grade ≥2 AEs occurred in 63 patients (43%), of whom 16 stopped treatment owing to the AEs.

### Examination of factors contributing to OS

**Figure 2:**
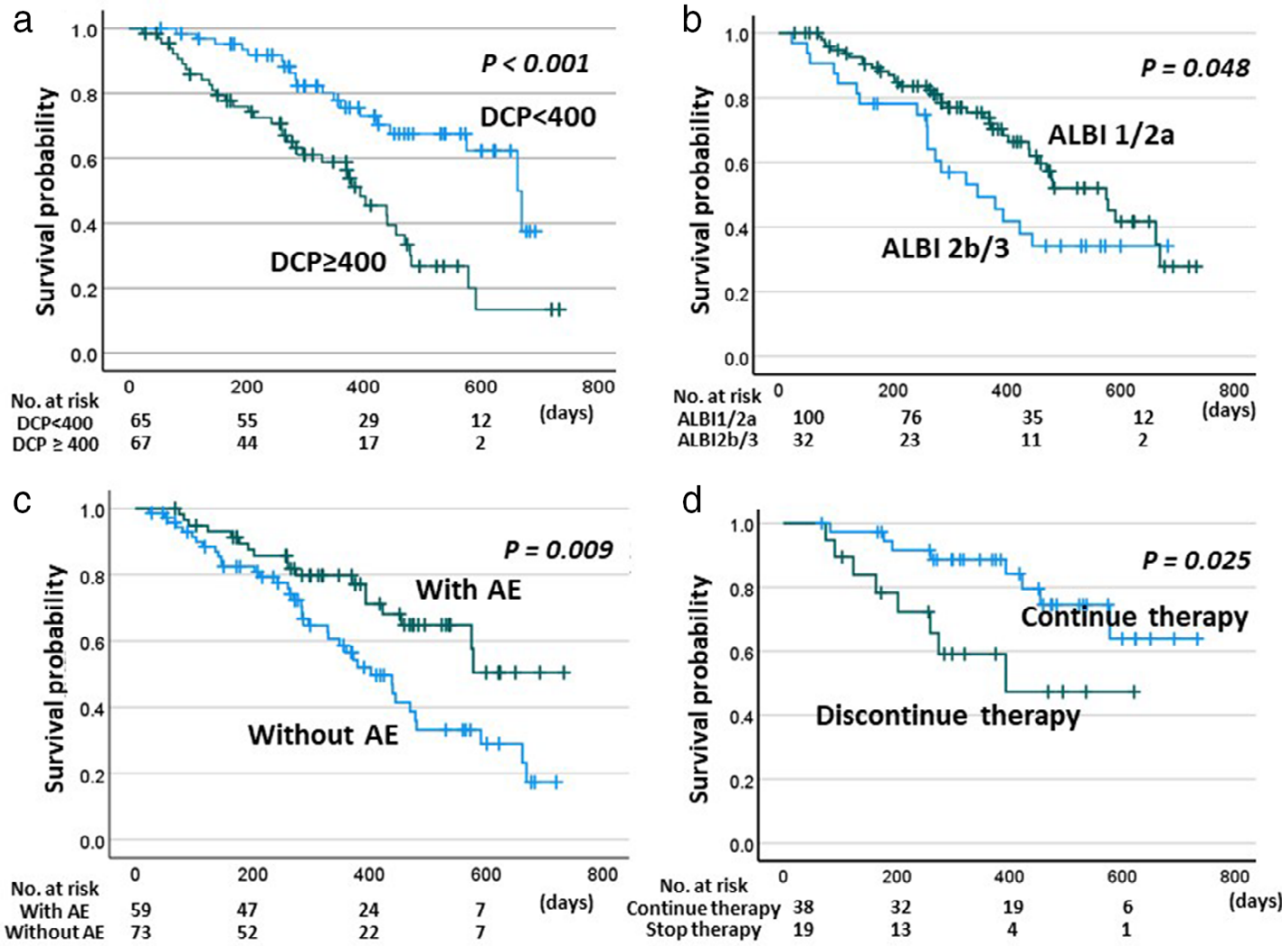
Kaplan–Meier plots of overall survival. Overall survival was determined according to the serum DCP level before therapy (a), albumin–bilirubin (ALBI) score before therapy (b), and occurrence of grade ≥ 2 adverse events after starting therapy (c). Overall survival in patients with grade ≥ 2 adverse events was also compared between patients who continued treatment and patients who discontinued treatment owing to adverse events (d). DCP, des‐gamma carboxy prothrombin; ABLI, albumin–bilirubin.

When we examined which factors contributed to OS, significant factors in the univariate analyses were hepatitis viral etiology (HR; 0.499, 95% CI; 0.290–0.859, *P* = 0.012), existence of varices prior to chemotherapy (HR; 1.902, 95% CI; 1.094–3.307, *P* = 0.023), pretreatment albumin‐bilirubin (ALBI) grade 1 or 2a (HR; 0.575, 95% CI; 0.330–0.992, *P* = 0.049), and high level of DCP (HR; 2.831, 95% CI; 1.619–4.951, *P* = 0.001) as pretreatment factors, together with the occurrence of grade ≥2 AEs (HR; 0.480, 95% CI; 0.273–0.842, *P* = 0.001) and achievement of CR/PR as best treatment response (HR; 0.217, 95% CI; 0.105–0.447, *P* = 0.001) as on/post‐treatment factors. After applying the stepwise selection, the factors that contributed to OS in the multivariable model were low DCP level before therapy, achievement of CR/PR, and the occurrence of grade ≥2 AEs (Table 2). Figure 2 shows the survival curves for these factors. There was a significant increase in survival in patients with a low DCP level before treatment (Fig. 2a), patients with ALBI 1/2a (Fig. 2b), and patients with grade ≥2 AEs (Fig. 2c; log‐rank test).

**Table 2:** Factors associated with overall survival

	Univariate HR (95% CI), *P* value	Multivariable model HR (95% CI), *P* value
Sex	0.747 (0.407–1.373), 0.348	
Age	1.006 (0.977–1.036), 0.693	
Virus	0.499 (0.290–0.859), 0.012*	0.562 (0.260–1.218), 0.144
Alcohol	1.038 (0.601–1.795), 0.893	
Varices	1.902 (1.094–3.307), 0.023*	1.302 (0.593–2.860), 0.510
Hypertension	0.933 (0.548–1.587), 0.797	
Diabetes	0.724 (0.373–1.405), 0.339	
Prior therapy	1.247 (0.629–2.472), 0.528	
Prior chemotherapy	1.325 (0.777–2.260), 0.301	
AFP > 400 ng/mL	1.416 (0.833–2.406), 0.199	
DCP > 400 mAU/L	2.831 (1.619–4.951), 0.001*	2.781 (1.394–5.545), 0.004*
Vessel invasion	1.680 (0.909–3.106), 0.098	
Extrahepatic metastasis	1.295 (0.722–2.322), 0.386	
ALBI 1/2a	0.575 (0.330–0.992), 0.049*	0.429 (0.221–0.831), 0.012*
Achievement of CR/PR	0.217 (0.105–0.447), 0.001*	0.244 (0.102–0.580), 0.001*
Post‐treatment	0.865 (0.508–1.472), 0.592	
Grade ≥2 AE	0.480 (0.273–0.842), 0.011*	0.458 (0.197–0.965), 0.035*
Discontinued treatment due to AE	1.016 (0.434–2.377), 0.971	

* Statistically significant.

HR, hazard ratio; CI, confidence interval; AFP, alpha‐fetoprotein; DCP, des‐gamma‐carboxy prothrombin; ALBI, albumin‐bilirubin; CR, complete response; PR, partial response; AE, adverse event.

### Examination of grade ≥2 AEs

**Figure 3:**
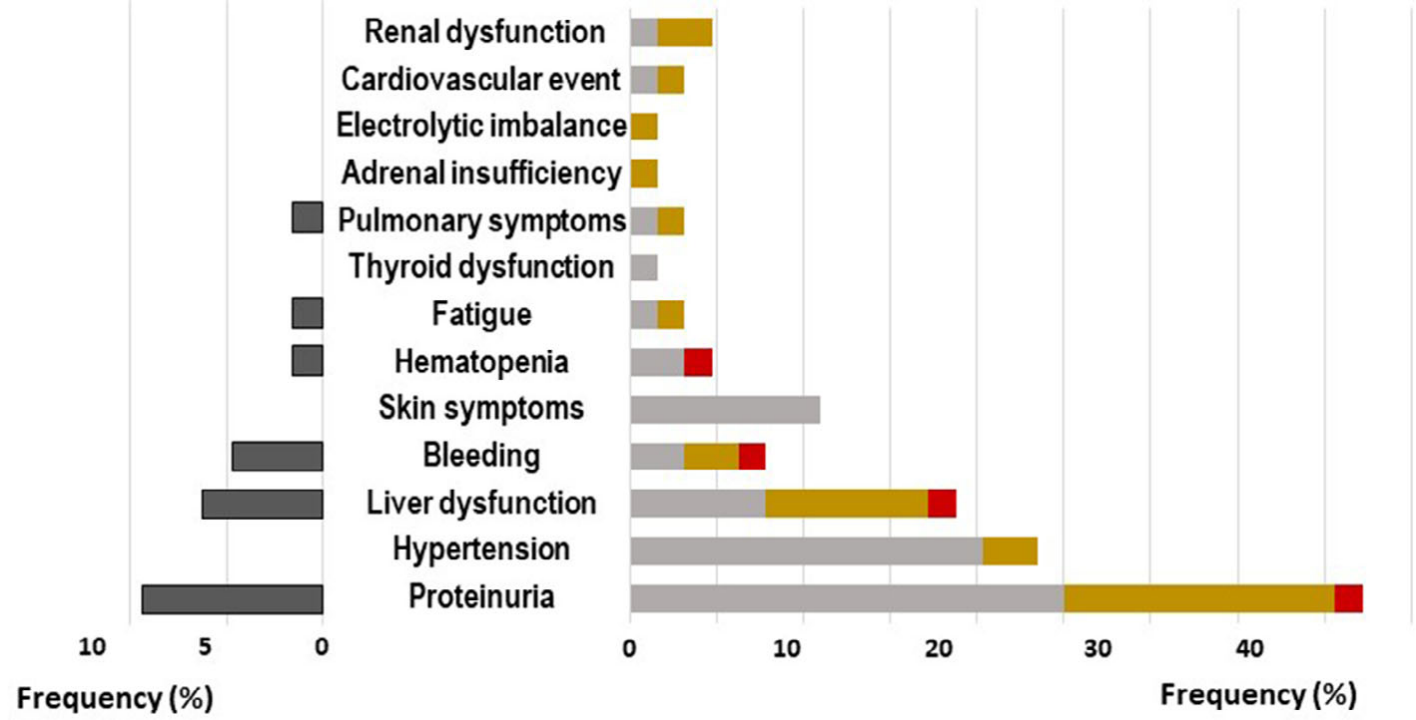
Breakdown of grade ≥2 adverse events. The right column shows the frequency of adverse events by grade, and the left column shows the frequency of discontinuations due to adverse events. 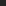 , treatment discontinued; 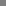 , grade 2; 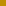 , grade 3; 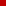 , grade 4.

Figure 3 shows the frequency of grade ≥2 AEs and the proportion of patients who discontinued treatment owing to AEs. The most frequent AE was proteinuria (27 patients, 42.2%), and 6 patients (9.4%) discontinued owing to proteinuria. Hypertension occurred in 15 patients (23.4%), but none led to treatment discontinuation. Liver dysfunction occurred in 12 patients (18.8%), and 4 patients (6.3%) discontinued. Other AEs included bleeding in 5 patients (7.8%), skin symptoms in 7 patients (10.9%), thrombocytopenia in 3 patients (4.7%), fatigue in 2 patients (3.1%), and lung injury in 2 patients (3.1%). By grade, 36 patients suffered grade 2 AEs, 23 suffered grade 3 AEs, and 4 suffered grade 4 AEs. Patients who could not continue treatment were 3 (8.3%) of those with grade 2 AEs, 9 (39%) of those with grade 3 AEs, and all patients with grade 4 AEs.

Log‐rank tests with the Kaplan–Meier method were performed for patients with grade ≥2 AEs. Figure 2d shows that the OS of patients who discontinued owing to AEs was significantly shorter than that of the other patients (*P* < 0.025).

## Discussion

In this study, low DCP level and ALBI 1/2a before therapy were expected to be predictive factors of efficacy. It was supportive that many factors have been reported to predict the efficacy of atezolizumab/bevacizumab combination therapy, including the ALBI score before treatment and changes in the AFP and DCP levels after the start of treatment.^10^, ^11^, ^12^ It is also reasonable that achievement of CR/PR is related to OS. On the other hand, our study also revealed that patients with AEs are involved in OS.

With regard to ICIs, which were introduced in recent years, the appearance of immune‐related AEs in patients with lung cancer or malignant melanoma indicates activation of the patient's immune response, which contributes to the antitumor effect and prolonged OS.^13^, ^14^, ^15^, ^16^ Regarding HCC, although the significance of immune‐related AEs has not been fully demonstrated, these events were shown to be associated with PFS.^7^ The results of this study support this concept. However, a survival benefit was not observed in patients who discontinued treatment owing to AEs. Therefore, it is important to continue treatment by reducing the dose or adjusting the duration of administration as appropriate.

In this study, hypertension, proteinuria, and bleeding, which are considered to be associated with bevacizumab, were the most frequent events, whereas immune‐related AEs of grade ≥2 were less frequent, occurring in 18 patients. Interstitial pneumonia occurred in two patients and led to treatment discontinuation in both patients. The complications of interstitial pneumonia can be severe, demonstrating the need for caution.^17^ Proteinuria with grade ≥2 was observed in 26 patients and resulted in treatment discontinuation in six of these patients. Because it was recently shown that changes in proteinuria early in treatment are associated with grade ≥2 proteinuria, physicians should be vigilant for early changes in proteinuria after starting treatment.^18^ Nevertheless, many patients with bevacizumab‐related AEs could continue atezolizumab as monotherapy. Thus, it is important to maintain the antitumor effects by continuing treatment.

Bevacizumab‐related bleeding was reported as a grade ≥2 AE in seven patients. The atezolizumab/bevacizumab combination therapy was discontinued in four patients with ruptured esophageal varices reported as grade ≥3 AEs and in one patient with cerebral hemorrhage. All of the patients included in this study underwent endoscopy and received prophylactic treatment prior to the start of therapy. However, in prior reports, atezolizumab/bevacizumab combination therapy was associated with worsening esophageal varices and a high risk of rebleeding in patients with a history of variceal rupture.^19^, ^20^, ^21^ Therefore, endoscopic examination is considered necessary during treatment, especially in high‐risk patients.

Currently, atezolizumab/bevacizumab combination therapy is often chosen as first‐line systemic chemotherapy for HCC, whereas lenvatinib and sorafenib are often used as second‐line therapy. Durvalumab combined with tremelimumab has also shown promising efficacy, and its use may be considered in the future in consideration of the patient's general condition and the risk of AEs.^22^

Limitations of this study include the fact that it was a multicenter study, there were no criteria for starting or changing chemotherapy, and switching to atezolizumab monotherapy was at the discretion of the primary care physician.

In conclusion, not only factors before therapy but also treatment response and the appearance of AEs are involved in OS for atezolizumab/bevacizumab combination therapy. Although the development of AEs also contributed to OS, appropriate management of AEs is important to avoid discontinuing treatment with this combination.

## Ethics statement

The study was carried out in accordance with the Declaration of Helsinki and was approved by the institutional review board of the Kobe University Graduate School of Medicine and by the institutional review boards of the participating hospitals (no. B200286).

## Patient consent

The need to collect informed consent from the patients was waived because this was a retrospective study. Information about this study was published in our institute, and patients could ask for their data to be withdrawn from the analysis.
